# Prospects for the Use of ATR Inhibitors to Treat Cancer

**DOI:** 10.3390/ph3051311

**Published:** 2010-04-28

**Authors:** Jill M. Wagner, Scott H. Kaufmann

**Affiliations:** Division of Oncology Research, College of Medicine, Mayo Clinic, 200 First St., S.W., Rochester, MN 55905, USA; E-Mail: wagner.jill@mayo.edu (J.M.W.)

**Keywords:** Replication checkpoint, ATR, ATM, chemotherapy, Chk1

## Abstract

ATR is an apical kinase in one of the DNA-damage induced checkpoint pathways. Despite the development of inhibitors of kinases structurally related to ATR, as well as inhibitors of the ATR substrate Chk1, no ATR inhibitors have yet been developed. Here we review the effects of ATR downregulation in cancer cells and discuss the potential for development of ATR inhibitors for clinical use.

## 1. Introduction

All cells have mechanisms to maintain the integrity of their genomes. Cell cycle checkpoint pathways have evolved to regulate cell cycle progression, DNA replication, and repair of damaged DNA [1,2]. The ataxia telengiectasia and Rad3-related (ATR) protein kinase is the apical kinase of one such checkpoint pathway. The best known role of ATR is its activation of checkpoint kinase 1 (Chk1) after replication fork stalling, leading to cell cycle arrest. A growing body of data, however, now shows roles for ATR far beyond the activation of Chk1. For example, ATR also phosphorylates numerous substrates in other DNA repair pathways. In addition, ATR plays a role in normal replication of undamaged DNA.

Many current cancer treatments, including chemotherapeutic agents and ionizing radiation, induce DNA damage and replication fork stalling, thereby activating cell cycle checkpoint pathways. A variety of studies have shown that this response is an important mechanism that helps cancer cells survive these treatments. These findings have prompted the development of agents targeting DNA-damage response signaling pathways. Although several inhibitors of Chk1 have been developed and have proceeded to clinical trials, no inhibitors of ATR have yet been developed.

In this review, we summarize current understanding of ATR signaling pathways. We also examine the effects of ATR downregulation in combination with chemotherapeutic agents and discuss the possibility of developing ATR inhibitors for clinical use.

## 2. The PIKK Family

**Figure 1:**
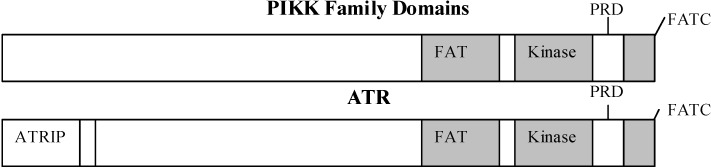
Structural domains of ATR in comparison to other PIKK family members. The shaded domains share the highest degree of sequence homology. ATRIP (ATR-interacting protein), FAT (FRAP, ATM, TRRAP), PRD (PIK regulatory domain), FATC (FAT domain at the carboxyl terminus).

ATR is a member of the phosphoinositide 3-kinase related kinase (PIKK) family [3,4]. PIKKs are large serine-threonine protein kinases that exhibit a high degree of sequence homology particularly in their kinase, FAT (named after the kinases FRAP, ATM, and TRRAP) and FATC domains. The PIKKs show a similar overall architecture (Figure 1). The kinase region is bordered by the FAT domain on the N-terminal side and the FATC domain on the C-terminal side. The FAT domain is part of a long N-terminal region predicted to fold into alpha-helical HEAT repeats, each 37-47 amino acids long. Although the functions of the FAT and FATC domains are not firmly established, it has been speculated they may be important for proper functioning of the kinase domain or for interactions with other proteins [5]. The PIK regulatory domain (PRD), located between the kinase and FATC domains, is a regulatory domain in at least ATR, ATM, and the mammalian target of rapamycin (mTOR), and is poorly conserved. The N-terminus, also poorly conserved, allows for interaction with accessory proteins. Electron microscopy structures, which have been reported for DNA-PKcs and ATM only, show that PIKKs assume a large globular conformation in which distant regions of the primary sequence can be brought together through folding [6,7].

## 3. Structure of ATR/ATRIP

A more detailed understanding of the structure of ATR and how it interacts with other proteins has emerged in recent years. ATR is a large kinase consisting of 2644 aa, with a molecular weight of 300 kD. ATR-interacting protein (ATRIP), the 85 kDa binding partner of ATR, binds to the N-terminus of ATR [8]. Because there is no difference in phenotypes that result from the loss of ATR or ATRIP (reviewed in ref. [9]), ATRIP should probably be considered a subunit of the ATR holoenzyme.

Some ATR-interacting proteins actually bind to ATRIP. For example, ATRIP binds to replication protein A (RPA) on single-stranded DNA [10]. This interaction occurs through binding of an acidic alpha helix in ATRIP with the basic cleft of an N-terminal OB-fold domain in the large RPA subunit [11]. In addition, the primary binding site for the topoisomerase binding protein I (TopBP1) activation domain is also within ATRIP [12]. Activation of ATR also requires the PRD domain, as mutations in this region prevent activation of ATR by TopBP1, causing severe checkpoint defects [12]. Five phosphorylation sites have been mapped on ATR/ATRIP, yet none is a clear early marker for ATR activation [13,14,15].

## 4. Comparison of ATR and ATM

A comparison of ATR and ATM shows structural as well as broad functional similarities. ATR shares considerable sequence homology with ATM. The FAT domain of ATR shares 23 of 43 amino acids with ATM; the kinase domain shares 35 of 55 amino acids; and a functionally undefined N-terminal region has 21 of 39 amino acids in common with ATM [16]. In addition to the sequence homology and similar structural architecture, ATR and ATM both prefer to phosphorylate serine or threonine residues followed by a glutamine [17,18]. These S/T-Q sites are clustered in some substrates but can be found singly in others. Although ATR and ATM each have specific substrates, there are some substrates common to both, including RPA [19] and BRCA1 [20].

ATR is essential for cell survival [21]. ATR^-/-^ cells develop widespread chromosome breaks and then undergo apoptosis. As a consequence, knockout of *Atr* in mice is embryonic lethal [21]. In contrast, even though ATM^-/-^ cells are hypersensitive to ionizing radiation and other agents that cause double-strand breaks (see below), ATM is not essential for cell survival. 

## 5. The ATR and ATM Checkpoint Pathways

**Figure 2:**
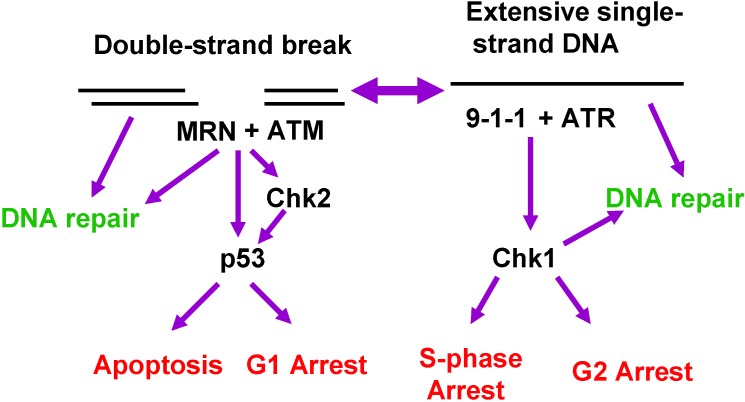
ATM and ATR signaling pathways. DNA DSBs generally activate ATM, which along with the Mre11-Rad50-NBS1 complex, can facilitate DNA repair or phosphorylate the Chk2 kinase leading to activation of p53 followed by G1 arrest or apoptosis. Alternatively, extensive regions of single-stranded DNA that result from the continued activity of helicases after replication forks are stalled (see Figure 3) activate the ATR pathway. ATR, together with the Rad9-Hus1-Rad1 complex can activate Chk1. Chk1 in turn phosphorylates Cdc25A and Cdc25C, which targets the former for degradation and the latter for sequestration, causing the cells to arrest in S or G2. Chk1 activation can also facilitate DNA repair.

ATR and ATM are the apical kinases for the two checkpoint pathways triggered by DNA damage. Chk1 and Chk2 are critical kinases downstream of ATR and ATM, respectively, playing roles in cell cycle arrest, DNA repair, or apoptosis (Figure 2).

**Figure 3:**
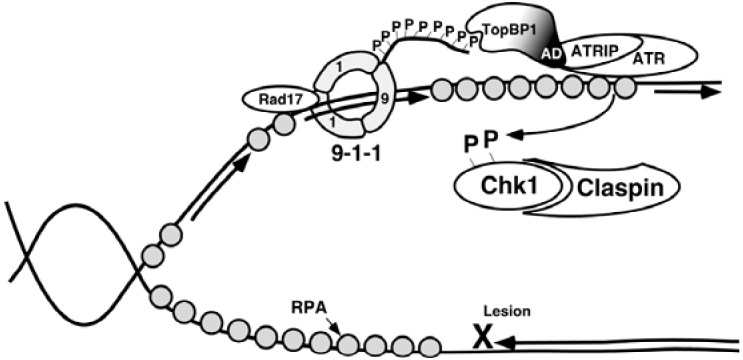
Model of the activation of ATR through Rad9 and TopBP1. See text for details. Modified from [27].

ATR and ATM are generally thought to respond to different types of DNA damage [22]. ATR is activated in response to ultraviolet light, certain chemotherapeutic drugs, hydroxyurea, and replication stress. When these agents cause polymerases to stall during replication at damage sites on DNA, helicases will continue to unwind the DNA, leading to long stretches of ssDNA [10]. Replication protein A (RPA) coats ssDNA. The ATRIP portion of the ATR-ATRIP complex then directly binds RPA through conserved binding regions. This binding of ATR-ATRIP to RPA-coated ssDNA does not in itself, however, activate ATR. Activation also requires the Rad9-Hus1-Rad1 (9-1-1) complex and topoisomerase binding protein I (TopBP1) (Figure 3). The 9-1-1 complex, a heterotrimeric ring similar in structure to the replicative sliding clamp PCNA, is loaded by Rad17 and the four small replication factor C (RFC) subunits, in an ATP dependent manner, onto RPA-coated ssDNA [23,24]. More specifically, 9-1-1 is preferentially loaded onto DNA with 5’-recessed ends, including stalled replication forks at sites of damage, recombination sites, and nucleotide-excision repair sites [25]. Next, the BRCT I and II domains of TopBP1 interact with phosphorylated serine^317^ on chromatin-bound Rad9 [26]. This brings the activation domain of TopBP1 in proximity to RPA-bound ATR, allowing the activation of ATR [26]. Although this interaction with TopBP1 increases the kinase activity of ATR toward its substrates [27], it is not known how this occurs. One possibility is the binding of ATR to the activation domain of TopBP1 changes the conformation of the kinase domain of ATR [28]. 

**Figure 4:**
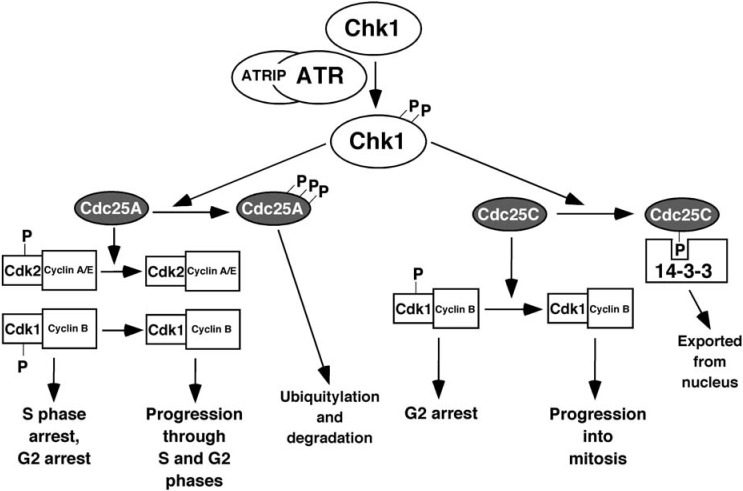
Chk1 mediates ATR-induced cell cycle arrest. ATR activates Chk1, which then phosphorylates and inactivates Cdc25A and Cdc25C. Phosphorylated Cdc25A is ubiquitylated and degraded, leaving Cdk2/cyclin complexes in their inactive form, resulting in S-phase and G2-phase arrest. Phosphorylated Cdc25C binds to 14-3-3 proteins and is exported from the nucleus, leaving Cdk1/cyclin B complexes in the inactive form, resulting in G2 arrest.

Among the many substrates phosphorylated by activated ATR, the most widely studied is Chk1 [29]. Another protein, claspin, serves as an adaptor to bring ATR and Chk1 together [30,31,32,33]. The Tim/Tipin complex may also serve to bring ATR and Chk1 together [34,35]. After ATR phosphorylates Chk1 on Ser^317^ and Ser^345^, activated Chk1 then phosphorylates many serine residues on the phosphatase Cdc25A (Figure 4), leading to its ubiquitylation and degradation [36]. As a result, Cdc25A is not available to remove inhibitory phosphorylations on Cdk1/cyclin B, causing cells to arrest in G2 phase of the cell cycle. Activated Chk1 can also phosphorylate Cdc25C, causing it to bind 14-3-3 proteins and be exported from the nucleus [37,38]. This also leads to G2 arrest, as the cytoplasmic Cdc25C cannot remove the inhibitory phosphorylations on nuclear Cdk1/cyclin B.

The phosphorylation of Chk1 by ATR can also lead to an intra-S phase checkpoint [39]. When DNA is damaged, ongoing processes that involve the DNA, such as replication, can lead to further damage. ATR signaling is critical for inhibiting the firing of new origins of replication [40,41,42], thereby diminishing this replication-associated damage. The regulation of replication origins by ATR signaling involves, at least in part, Chk1-mediated phosphorylation of Cdc25A, which then cannot de-phosphorylate and activate the Cdk2/cyclin A complex required for S-phase progression [36]. In both yeast and mammalian cells, ATR acting through checkpoint kinases slows elongation of replication forks [43]. The ATR mediated S-phase checkpoint also directly inhibits the rereplication of DNA, *i.e.*, replication more than once in a cell cycle, as measured by the percentage of cells with > 4N DNA [44]. 

In contrast to the ATR pathway, the ATM pathway (Figure 2) is activated after DNA double-strand breaks (DSBs), which may result from ionizing radiation or certain chemotherapeutic drugs. ATM, activated by its interaction with the Mre11-Rad50-NBS1 complex (MRN), phosphorylates a variety of substrates [45], most notably Chk2. Once activated by this phosphorylation, Chk2 not only phosphorylates Cdc25A and Cdc25C as described above, but also the transcription factor p53, which activates the gene encoding p21, an inhibitor of the Cdk2/cyclin A and Cdk2/cyclin E complexes. This sequence of events constitutes the G1 checkpoint, which prevents cells with damaged DNA from entering S phase. The activation of p53 may also lead to apoptosis of cells if damage is beyond repair [46,47].

## 6. Stimuli that Activate the ATR-Chk1 Pathway

### 6.1. DNA damage

ATR can be activated by a variety of types of DNA damage [48]. Endogenous DNA damage may result from the metabolic production of reactive oxygen species. As indicated above, exogenous damage that activates ATR signaling can be caused by ultraviolet light, hydroxyurea, replication stress, or chemotherapeutic agents. For example, the chemotherapeutic drugs gemcitabine and cytarabine act as nucleoside analogs that are incorporated into DNA during replication; however, upon incorporation, they cause replication fork stalling [49]. In all of these cases, the ATR-Chk1 pathway plays a critical role in the response to the damage [50]. Single-stranded DNA (ssDNA) is the common intermediate responsible for activation of ATR by these divers agents [10]. Single-strand breaks may, however, lead to DSBs, which then activate ATM. Conversely, DSBs may be processed to ssDNA. Thus, there is crosstalk between the ATR and ATM pathways, and there is not always a clear distinction between agents that activate each pathway.

### 6.2. Fragile sites and repetitive DNA sequences

Aside from replication stress due to lesions caused by DNA damaging agents, there are other situations where replication may be slowed or stalled. These situations include the replication of fragile sites and repetitive sequences.

Fragile sites are large chromosomal regions that replicate late during S phase and are prone to gaps and breaks, which can be seen in metaphase chromosomes during karyotyping [51]. Common fragile sites have been implicated in cancer. The specific nature of the DNA at these common fragile sites is not entirely clear but may involve changes in flexibility of the DNA, nucleotide content, or the presence of repeated elements at the sites.

Common fragile sites are normally stable in cultured human cells. However, following treatment with replication inhibitors such as aphidicolin, these sites show an increase in the number of sister chromatid exchanges, translocations, and deletions [51]. “Expression” of fragile sites can be measured by counting the number of gaps/breaks at a known fragile site. It is not clear whether ATR is recruited when the replication fork encounters a fragile site. However, the absence of ATR or Chk1 causes an increase in the number of gaps/breaks at common fragile sites after treatment with aphidicolin [52,53]. Similarly, mice with mutations in ATR have increased expansion rates of repeats associated with Fragile X syndrome [54]. Replication slow zones in yeast, which are analogous to fragile sites, likewise require the yeast ATR homolog Mec1 for stability [55].

Repetitive DNA sequences can form secondary structures such as hairpins that can also interfere with progression of replication forks [56]. In *S. cerevisiae*, CAG repeats form secondary structures that block replication forks. These regions are unstable when Mec1 or Rad53 (Chk2 ortholog) is absent; repeat contraction and breakage then occur at greater frequency [57]. Although these observations suggest that ATR signaling is important for replication and stabilization of forks at fragile sites and repetitive sequences, exactly how ATR and Chk1 regulate this remains to be determined.

### 6.3. Shortened telomeres

Critically short telomeres also activate a DNA damage response. Telomeres are structures on the ends of chromosomes that facilitate replication and protect the ends from degradation. In cells lacking telomerase, a single short telomere is enough to recruit checkpoint proteins [58]. An uncapped shortened telomere is thought to be recognized as a DSB by DNA damage response proteins. In senescent human fibroblasts, for example, the shortened telomeres directly associate with many DNA damage proteins, as evidenced by nuclear foci of phosphorylated histone H2AX, 53BP1, MDC1, and NBS1 [59]. Although initial recognition of a DSB is through the ATM pathway, the subsequent processing of DNA ends can activate the ATR pathway. Thus, senescent human fibroblasts contain activated Chk1 as well as Chk2 [59].

DNA damage response proteins are not only responsible for recognizing short telomeres, but are also important in maintaining normal telomeres. In *S. cerevisiae*, Mec1 mutants (the ortholog of mammalian ATR) have shortened telomeres compared to wild-type, and yeast lacking both Mec1 and Tel1 (ATM ortholog) experience progressive telomere erosion because their telomeres fail to recruit telomerase [60]. The ATR pathway has been more specifically implicated in telomere maintenance by the finding that the 9-1-1 complex is an integral component of the telomere in human and mouse cells [61]. Moreover, mouse cells deficient in the Hus1 portion of the complex have much greater telomere shortening than wild-type cells [61].

## 7. ATR Substrates

Results from a large-scale proteomic analysis of proteins phosphorylated in response to DNA damage on consensus sites recognized by ATR and ATM identified over 700 substrate proteins with more than 900 phosphorylation sites [62]. Although many of these proteins are substrates of ATM, there are also a large number of potential ATR substrates in this set. This set of proteins is highly interconnected and describes a much more complicated network for the DNA damage response than was previously understood.

The most thoroughly studied ATR substrate is the kinase Chk1. As discussed above, Chk1 is activated when ATR phosphorylates it on Ser^317^ and Ser^345^. Once phosphorylated, Chk1 is released from chromatin to phosphorylate its own substrates, leading to diminished replication and slowed cell cycle progression, thereby allowing time for repair to occur. Additionally, ATR and Chk1 help regulate DNA replication in undamaged cells. Specifically, ATR and Chk1 regulate the timing of the firing of origins during normal, unperturbed replication [63]. In the absence of damage, loss of Chk1 activity results in the frequent stalling and, possibly, collapse of active forks as well as activation of adjacent, previously suppressed origins [64]. 

Additional ATR substrates involved in replication and stabilization of stalled forks include the replication factor C complex, RPA1 and RPA2, and the MCM2-7 complex [65,66,67,68]. Phosphorylation of these substrates can lead to inhibition of replication, which goes along with the checkpoint function of ATR, or can lead to the restarting of replication, *i.e.*, recovery from replication inhibition. For example, MCM2 phosphorylation by ATR recruits Polo-like kinase 1 (Plk1), which then promotes recovery of DNA replication [69].

Additional ATR substrates that play roles in various checkpoint and repair pathways have been identified. BRCA1 (breast cancer suppressor protein 1) plays a role in both non-homologous end joining (NHEJ) and homologous recombination (HR). Although the exact role of BRCA1 in these processes is not clear, it is phosphorylated by ATR following damage by IR or hydroxyurea [70]. Notably, BRCA1 is also a substrate for ATM, which phosphorylates it following IR.

WRN and BLM are two additional ATR substrates that are thought to play critical roles during replication. WRN, the protein missing in Werner's syndrome, an autosomal recessive disorder characterized by genomic instability, is phosphorylated through an ATR/ATM dependent pathway in response to replication blockage [71]. Bloom helicase (BLM), the protein mutated in Bloom syndrome, which is characterized by predisposition to almost all forms of cancer, is phosphorylated by ATR at Thr^99^ after replication stress. This phosphorylation is critical for BLM to interact with and colocalize with 53BP1 [72]. BLM and WRN are thought to act at stalled replication forks to prevent recombination and nuclease action at the site [73,74,75].

ATR also participates in regulation of the Fanconi anemia repair pathway, a pathway specifically implicated in repair of DNA cross-links. ATR phosphorylates FANCD2 on Thr^691^ and Ser^717^, thereby promoting FANCD2 monoubiquitination and enhancing cellular resistance to DNA cross-linking agents such as cisplatin [76].

## 8. Rationale for Inhibiting ATR in Cancer

Because the ATR-Chk1 checkpoint pathway serves to insure cell survival after replication stress, a normal and robust checkpoint is thought to be a mechanism of resistance to chemotherapy. As a result, ATR-Chk1 pathway components are considered promising therapeutic targets. In particular, inhibition of ATR-Chk1 pathway components could potentially enhance the effectiveness of replication inhibitors. A potential advantage of sensitizing cells in this way would be the use of lower doses of the replication inhibitor, thus reducing toxicity to hematologic, gastrointestinal, and other organ systems, if the normal cells are not sensitized to the same extent.

A variety of observations suggest that checkpoint inhibition might indeed selectively sensitize cancer cells. It has been known for two decades that most tumor cells are deficient in the G1 checkpoint. For example, many cancers have mutations in p53 or other components of the p53 pathway, leading to reliance on the S and G2 checkpoints to arrest the cell cycle and provide for repair and survival [77,78,79]. Inhibition of the S and G2 checkpoints may then preferentially kill these p53 deficient tumor cells. This is illustrated by the effects of caffeine, a PIKK inhibitor, which preferentially sensitizes p53 mutant cells to ionizing radiation [80,81]. Alternatively, one or more of the other components of checkpoint responses may be mutated or impaired in cancer cells. Here, too, inhibition of the remaining checkpoints is likely to have greater deleterious effect on cancer cells than on normal cells because of intact function of all of the checkpoints in normal cells.

Specificity for cancer cells may also be insured by the fact that untransformed cells have more robust S and G2 checkpoints than tumor cells, as illustrated in recent work on the role of checkpoints at replication forks stalled by hydroxyurea treatment [82]. In normal cells, both the Chk1 pathway and several Chk1-independent pathways are activated after replication fork stalling. The Chk1-independent mechanisms in untransformed cells include activation of p38, which collaborates with Chk1 to prevent mitotic entry. Down-regulation of cyclin B1 promoter activity also occurs, independent of Chk1 or p38, after replication fork stalling in untransformed cells. Tumor cells, in contrast, rely entirely on the Chk1 pathway for the needed response, perhaps because they lack one or more additional parallel pathways that, in untransformed cells, ensure a replication checkpoint response in the absence of Chk1. Thus, inhibition of Chk1 along with hydroxyurea enhances the killing of tumor cells but not untransformed cells [82].

Collectively, these studies provide strong support for the use of inhibitors of the ATR-Chk1 pathway in combination with DNA damaging chemotherapies.

## 9. Inhibition of ATR in Cancer Therapy

### Known inhibitors of ATR

A variety of strategies are used by investigators to inhibit ATR in the preclinical setting. It is important to emphasize that these strategies have varying degrees of specificity. Some of the chemical inhibitors used in preclinical studies are nonspecific and will also inhibit ATM and possibly other kinases. For example, caffeine, one of the earliest inhibitors in use, appears to sensitize tumor cells to ionizing radiation (IR) and other genotoxic agents by inhibiting the catalytic activity of both ATR and ATM [83]. Not only does caffeine inhibit both of these PIKKs in A549 lung cancer cells after IR, but the S and G2 arrest normally seen after IR is blocked by caffeine at concentrations similar to those that block ATR and ATM catalytic activity in kinase assays [83].

The fungal metabolite wortmannin has also been used in studies involving inhibition of ATR. Like caffeine, wortmannin inhibits multiple PIKKs. In particular, wortmannin also inhibits ATM and hSMG-1 [84].

More recently, the molecule schisandrin B was identified as a somewhat selective ATR inhibitor by screening herbal extracts and ingredients [85]. Isolated from the fruit of *Scisandra chinesis*, which is commonly used in traditional Chinese medicine for treating hepatitis and myocardial disorders, schisandrin B showed an inhibitory effect in UV treated cells but not in IR treated cells. In particular, schisandrin B inhibited UV-induced Chk1 phosphorylation and sensitized A549 lung adenocarcinoma cells and AT2KY fibroblasts to UV treatment. Although ATR kinase activity *in vitro* was significantly decreased by schisandrin B, ATM kinase activity also diminished, albeit at much higher concentrations. Thus, schisandrin B seems to have some specificity for ATR, although it will also affect ATM at higher concentrations.

In view of this propensity for small molecules to inhibit ATR homologs as well as ATR itself, the use of PIKK inhibitors to elucidate the roles of ATR can be confusing or misleading. As a result, other strategies are often employed in an attempt to specifically diminish ATR activity. ATR siRNA or shRNA are widely used in studies of ATR signaling. Clear demonstration of the extent of ATR knockdown by Western blot can be a problem in some studies, however, due to the large size of ATR and poor quality of some commercial antibodies. 

As an alternative approach, some groups have overexpressed constructs encoding kinase-dead ATR [44,48,86], which has been shown to inhibit the action of wild-type ATR in a dominant negative fashion. Another group recently examined ATR inhibition in combination with chemotherapeutic drugs by constructing “DLD1-ATR-Seckel” cells, which are ATR mutant knock-in cells that express very low levels of ATR [87].

## 10. Sensitization of Cancer Cells to Chemotherapeutic Drugs by ATR Inhibition

Inhibition of checkpoint pathways is currently being investigated as a way to broaden the therapeutic window of anticancer agents such as antimetabolites, alkylating agents, platinating agents, and topoisomerase inhibitors. The use of ATR siRNA and other molecular techniques described in the preceding section, as well as the current clinical use of Chk1 inhibitors, give a glimpse of the potential efficacy of ATR inhibitors.

### 10.1. Antimetabolites

Gemcitabine, an antimetabolite used in the treatment of pancreatic, ovarian, and non-small cell lung cancers, provides an illustrative example of the potential utility of combination therapy with ATR inhibitors. Gemcitabine inhibits DNA replication through (i) inhibition of ribonucleotide reductase and (ii) acting as a nucleoside analog that is incorporated into the DNA and leads to chain termination [88]. As a consequence of both of these effects, gemcitabine activates the ATR-Chk1 pathway [89]. Moreover, downregulation of ATR, Chk1, or Rad9 sensitizes a variety of cells to gemcitabine [89,90]. The drug XL9844 (Exelixis, Inc.), which was developed as a specific inhibitor of Chk1 and Chk2, substantially enhances the cytotoxic effects of gemcitabine in multiple cell lines. Further, in a PANC-1 pancreatic cancer cell xenograft model, XL9844 significantly enhanced gemcitabine antitumor activity [91].

A second antimetabolite that is a candidate for use in combination with ATR-Chk1 pathway inhibitors is cytarabine (cytosine arabinoside), a cytosine analog used in the treatment of acute leukemia and other hematological disorders. Like gemcitabine, cytarabine inhibits replication and activates the ATR-Chk1 pathway. Downregulation of ATR, Rad9, or Chk1 also sensitizes cells to cytarabine [32,92].

A third antimetabolite that may be rendered more effective in combination with inhibitors of the ATR-Chk1 pathway is 5-fluorouracil (5-FU). 5-FU is used in the treatment of colon, gastric, head and neck, and other cancers. In a study examining the potential consequences of ATR or Chk1 mutations in cells with mismatch repair deficiency, Jardim *et al*. [93] produced HCT116 colon cancer cells in which ATR or Chk1 levels were stably knocked down to 50% of wild type levels. In these cells, the combination of ATR or Chk1 inhibition and mismatch repair deficiency resulted in enhanced 5-FU sensitivity. Sensitization to 5-FU through inhibition of Chk1 was also demonstrated by Ganzinelli *et al*. using inducible Chk1 siRNA clones [94]. Interestingly, the sensitivity to 5-FU was greater in p53-deficient colon cancer cells [94]. In contrast, siRNA inhibition of ATR or Chk1 by another group did not sensitize colon cancer cells to 5-FU (LM Karnitz, personal communication), illustrating the importance of cellular context in studying combinations of DNA damaging agents and pathway inhibitors.

### 10.2. Platinating agents

Platinating agents are another class of chemotherapeutic drugs that have been examined for use in combination with inhibitors of the ATR-Chk1 pathway. These agents, which include cisplatin, carboplatin, and oxaliplatin, are used in the treatment of ovarian, bladder, testicular, gastrointestinal, and other cancers. These agents are cytotoxic due to their ability to covalently bind to DNA and form intra- and inter-strand crosslinks that block replication and lead to lethal DSBs. Inhibition of ATR using siRNA was recently shown to sensitize a variety of cells to cisplatin, carboplatin, and oxaliplatin [95]. Unexpectedly, Chk1 inhibition did not sensitize these cells to the same platinating agents [95]. In a different study, expression of a kinase-dead ATR allele, which acts as a dominant negative, markedly sensitized GM847 SV40 transformed fibroblast cells to cisplatin, but not to oxaliplatin [96]. The difference in sensitivity to these two platinum agents may depend on the cellular context, although the mechanism for this is not currently understood. 

### 10.3. Alkylating agents

Alkylating agents are also potential candidates for use in combination with inhibitors of the ATR-Chk1 pathway. These agents add an alkyl group (C_n_H_2n+1_) to the guanine base of DNA, thereby interfering with DNA replication. Alkylating drugs currently used in chemotherapy include mitomycin C (MMC), temozolomide (TMZ) and nitrogen mustards such as melphalan. The use of checkpoint inhibitors in combination with MMC was investigated in a study of BCR/ABL-positive leukemia cells [97]. These cells accumulated more DNA DSBs than normal counterparts after treatment with MMC or cisplatin. Leukemia cells could repair these lesions more efficiently than normal cells and eventually survive. The increase in drug-induced DSBs in leukemia cells was associated with higher activity of ATR kinase and higher levels of phosphorylated Chk1. Inhibition of ATR by caffeine or Chk1 by the indolocarbazole inhibitor SB218078 sensitized BCR/ABL leukemia cells to MMC in clonogenic assays. This effect was associated with the abrogation of the S and G2/M cell cycle arrest after MMC treatment.

Inhibition of the ATR-Chk1 pathway together with TMZ treatment was investigated in a study examining the link between TMZ-induced modulation of Akt function and activation of ATR and ATM signaling pathways [98]. Colon cancer cells were sensitized to TMZ by siRNA to ATR, but not ATM. Interestingly, ATR was shown to be an upstream activator of Akt in the response to TMZ. Inhibition of ATR prevented Akt activation, thereby increasing cell sensitivity to TMZ. Another study by this same group implicated ATR in survival after TMZ treatment in mismatch repair proficient cells, but not in mismatch repair deficient cells [99].

The inhibition of ATR in combination with melphalan was investigated using two cell lines expressing a kinase-dead ATR. Cells expressing the kinase-dead ATR were more sensitive to melphalan than their wild-type counterparts, as measured by colony formation assays [96].

### 10.4. Topoisomerase poisons

ATR-Chk1 pathway inhibitors may also increase the effectiveness of drugs that target DNA topoisomerases. Topoisomerases are nuclear enzymes that catalyze the breaking and rejoining of the phosphodiester backbone to facilitate the unwinding of DNA during replication and transcription. Topoisomerase poisons block the religation step in this process, generating protein-linked single- and double-strand DNA breaks. Rad9, ATR, and Chk1 have all been shown to be important in survival of cells after damage caused by topoisomerase poisons. Colony forming assays using HeLa and U2OS cells demonstrated that down-regulation of ATR or Chk1 by siRNA sensitized cells to the topoisomerase I inhibitors camptothecin and SN-38 (the active metabolite of irinotecan). In contrast, down-regulation of ATM and Chk2 had minimal effect [33]. Similarly, expression of a dominant-negative ATR allele sensitized human fibroblasts to topotecan and camptothecin [100]. Consistent with these results, *Rad9* deletion also sensitized embryonic stem cells to treatment with camptothecin or etoposide [32]. 

### 10.5. A view based on ATR mutant cells

The role of ATR in resistance to DNA damaging agents was also investigated using ATR mutant knock-in cells that express very low levels of ATR [87]. The parent DLD1 colon carcinoma cells and the ATR mutant knock-in cells, termed “DLD-ATR-Seckel” cells, were treated with one of several different agents and assayed for survival in clonogenic assays. DLD-ATR-Seckel cells were strikingly sensitized to alkylating agents, including cisplatin, MMC and cyclophosphamide. Moderate sensitization was observed in cells treated with 5-FU, gemcitabine, or hydroxyurea. DLD-ATR-Seckel cells were not hypersensitive to taxol (a microtubule disruptor) or to TRAIL (an apoptosis-inducing ligand). Interestingly, in this same study, a nonspecific PIKK inhibitor actually increased clonogenic survival after 5FU treatment.

In summary, a growing body of data demonstrates the effectiveness of inhibiting the ATR pathway in combination with several classes of chemotherapeutic agents. With some of these chemotherapeutic drugs, e.g. gemcitabine, the inhibition of either ATR or Chk1 sensitizes cells to the drug. In these situations, the inhibition of Chk1 is sometimes as effective as ATR inhibition, but most often cells are sensitized to a lesser degree by Chk1 inhibition. Furthermore, there are also drugs like cisplatin where ATR downregulation or inhibition is effective in sensitizing but Chk1 downregulation or inhibition is not. These differences presumably reflect the action of ATR on a broad array of substrates and processes rather than on Chk1 alone.

## 11. Potential Problems with ATR Inhibition

Despite the promising results in cell lines, the clinical use of ATR inhibitors presents several potential problems. The most obvious concern is that *ATR* is an essential gene, and knockout of *Atr* in mice is embryonic lethal [21,101]. This raises the concern that inhibition of ATR may be lethal to normal cells. However, knockout of *Chk1* is also embryonic lethal in mice [29,101], yet small molecule Chk1 inhibitors are now being used in clinical trials in combination with chemotherapeutic drugs.

A second concern is that ATR regulates the firing of origins of replication in S phase of every cell. As a consequence, replication may not proceed properly without ATR. Also, errors in replication occur regularly in normal cells. It is not known whether cells can fully repair replication errors if ATR is not functional or is only active at low levels.

A third concern is that ATR inhibition may also increase breaks at fragile sites in the DNA of normal cells. These fragile sites may be especially prone to breakage after treatment with a drug that causes replication stress together with an inhibitor of ATR

A fourth issue involves the relationship between ATR and ATM signaling. As mentioned above, current evidence suggests significant crosstalk between these two kinases. Inhibition of ATR may affect the ability of the ATM pathway to function optimally. This could be harmful to normal cells if they are also exposed to DNA damaging agents. Additionally, lack of optimal ATM signaling through p53 could allow cancer cells to avoid apoptosis after treatment with DNA damaging drugs. However, the fact that many cancers are defective in p53 or another protein in the ATM-p53 pathway argues that ATR inhibition may not be a problem with regard to its effect on ATM and p53.

## 12. Clinical Trials Using Chk1 Inhibitors

Although specific ATR inhibitors have not yet been described, several inhibitors of checkpoint kinases have been developed to date. These include UCN-01, AZD7762, XL844, PF-00477736, CBP501, and SCH 900776 (Table 1). All of these drugs inhibit both Chk1 and Chk2, except SCH 900776, which is reportedly specific for Chk1. Of the remaining five inhibitors, PF-00477736 is the most selective for Chk1 (approximately 100-fold). XL844 is also 10-fold more selective for Chk1, while UCN-01, AZD7762, and CBP501 inhibit Chk1 and Chk2 to a similar degree. 

Results of clinical trials of UCN-01 in combination with cisplatin [102], carboplatin [103], topotecan [104], irinotecan [105], and fluorouracil [106] have been published. Partial responses were seen in 10% of patients in the topotecan + UCN-01 trial and in one of ten patients in the cisplatin + UCN-01 trial. No objective responses were seen in the trials of UCN-01 with carboplatin, irinotecan, or fluorouracil. Clinical trials of all other Chk1 inhibitors are currently ongoing, and publication of results from these trials is awaited with interest.

If clinical trials of these checkpoint inhibitors have limited success, targeting ATR might be a logical next step. Because ATR has functions beyond the phosphorylation of Chk1, inhibition of ATR may be more effective in enhancing the killing of cancer cells than the Chk1 inhibitors now in clinical trials. In the near future, we should learn whether inhibiting Chk1 increases the clinical antitumor efficacy of certain agents. In addition, questions regarding toxicity of combination therapies with checkpoint inhibitors and the risk of secondary cancers should be answered to some degree. Thus, these results could help guide future trials with ATR inhibitors.

**Table 1:** Clinical trials of checkpoint inhibitors in combination with DNA damaging agents or other drugs.

Inhibitor	Combination	Tumor types	Phase	Status	Trial identifier
UCN-01	Irinotecan	Solid	I	Completed	NCT00047242
		Solid or triple			
		negative breast	I	Recruiting	NCT00031681
	Topotecan	Ovarian	I	Completed	NCT00072267
			II	Completed	
		Small cell lung	II	Active	NCT00098956
	Gemcitabine	Pancreatic	I	Completed	NCT00039403
	Cisplatin (2)*	Solid	I	Completed	NCT00012194,
					NCT00006464
	Carboplatin	Solid	I	Completed	NCT00036777
	Fluorouracil	Pancreatic	II	Completed	NCT00045747
		Solid	I	Completed	NCT00004059
	Fluorouracil and Leucovorin	Solid	I	Completed	NCT00042861
	Prednisone	Solid or lymphoma	I	Completed	NCT00045500
	Perifosine	Leukemia	I	Recruiting	NCT00301938
	Fludarabine	Lymphoma or leukemia	I	Completed	NCT00019838
		Lymphoma or leukemia	II	Active	
	Cytarabine	Leukemia	I	Active	NCT00004263
AZD7762	Gemcitabine (2)*	Solid	I	Recruiting	NCT00413686,
					NCT00937664
	Irinotecan	Solid	I	Recruiting	NCT00473616
					
XL844	Gemcitabine	Solid	I	Ongoing	NCT00475917
					
PF-0477736	Gemcitabine	Solid	I	Recruiting	NCT00437203
					
CBP501	Cisplatin	Solid	I	Active	NCT00551512
	Cisplatin and Pemetrexed	Solid	I	Recruiting	NCT00942825
		Malignant pleural			
		Mesothelioma	II	Active	NCT00700336
SCH 900776	Gemcitabine	Solid or lymphoma	I	Recruiting	NCT00779584
	Cytarabine	Acute leukemia	I	Recruiting	NCT00907517

* Two separate studies with the same combination, tumor types, phase and status. Data from http://www.clinicaltrials.gov.

## 13. Prospects for Development of ATR Inhibitors

### 13.1. Issues in the design of small molecule ATR inhibitors

In view of the greater effects of ATR knockdown, particularly in the case of platinating agents, the development of specific ATR inhibitors remains an interesting possibility. As described in this section, however, the identification and development of selective ATR inhibitors is not without difficulty.

Kinase inhibitors are grouped into four types [107]. Type 1 inhibitors constitute the majority of ATP-competitive inhibitors and recognize the active conformation of the kinase, a conformation that normally enables phosphotransfer. By contrast, type 2 kinase inhibitors recognize the inactive conformation of the kinase. Both type 1 and 2 inhibitors have the ability to induce dramatic conformation changes in their target kinase.

Use of a type 1 inhibitor of ATR poses a significant risk of inhibition of ATM and other PIKK family members because of the high degree of homology in their kinase domains. Inhibition of these additional kinases may increase toxicity. Additionally, inhibition of the pathways controlled by other PIKK kinases could potentially counteract the inhibitory effect on ATR.

The third type of kinase inhibitors binds outside the ATP-binding site—at an allosteric site—and modulates kinase activity in an allosteric manner. Inhibitors belonging to this category tend to exhibit the highest degree of kinase selectivity because they exploit binding sites and regulatory mechanisms that are unique to a particular kinase. CI-1040, for example, is an allosteric inhibitor that inhibits MEK1 and MEK2 by occupying a pocket adjacent to the ATP binding site [108]. Development of an allosteric ATR inhibitor could avoid inadvertent inhibition of other PIKK family members, including ATM, if an ATR-specific site could be targeted. Targeting a site on ATRIP might also be a way to specifically inhibit ATR in an allosteric manner. 

A fourth type of kinase inhibitor forms an irreversible, covalent bond to the kinase active site, most frequently by reacting with a nucleophilic cysteine residue [109]. Examples of this type are HKI-272, an irreversible Her2 kinase inhibitor [110], and CL-387785, an irreversible epidermal growth factor receptor kinase inhibitor [111]. Although ATR could be targeted for inhibition by this approach, many drug developers are concerned about the potential for toxicity by these irreversible inhibitors if unanticipated targets were covalently modified by these inhibitors. 

### 13.2. Approaches for the development of ATR inhibitors

Small molecule inhibitors that target DNA-PK [112], ATM [113], and mTOR [114] have been developed. In contrast, a specific small molecule inhibitor of ATR has yet to be reported. This might reflect several hurdles.

First, ATR is a very large protein (2644 aa) and even larger when ATRIP is included. The large size could make it difficult to express in bacteria for subsequent use in kinase assays in a high-throughput screen. Because ATRIP is a required subunit of ATR, it may also be necessary to express ATR complexed to ATRIP in order to develop a high throughput assay.

Second, important structural information is lacking. No ATR crystal structure has been published to date. It may be easier and sufficient to crystallize a portion of the molecule, e.g., the kinase domain. However, the kinase domain will not contain information about allosteric binding sites required for development of a type 3 inhibitor.

A third hurdle is the difficulty in fully activating ATR *in vitro*. To screen for or test the effectiveness of inhibitors, one would ideally like an *in vitro* assay system using the active form of ATR. However, full activation requires several other molecules, including ATRIP, Rad9, TopBP1, and possibly RPA-coated single stranded DNA.

Given these difficulties with assaying ATR activity *in vitro*, perhaps the best approach would be to use a cell-based assay system to test potential inhibitors of ATR. In this situation ATR could be activated in cultured cells with a replication inhibitor such as hydroxyurea or aphidicolin. The cells could then be treated with potential ATR inhibitors. A logical readout for this type of assay would be the activating phosphorylation of Chk1 on Ser^317^ or Ser^345^, which could be detected as a cellular fluorescent signal using antibody to phosphorylated Chk1 and secondary fluorescent antibodies. A cell-based assay such as this could potentially be adapted for high throughput analysis of candidate ATR inhibitors.

## 14. Conclusions

Our understanding of the ATR-Chk1 signaling pathway has greatly increased over the past several years. A much broader role for ATR has also emerged, including functions in different DNA repair pathways. However, with hundreds of potential substrates of ATR identified by proteomics analysis, much work remains to thoroughly understand the consequences of ATR signaling.

The near future will provide results of the first clinical trials of checkpoint inhibitors in combination with a variety of chemotherapeutic agents. These trials use inhibitors of Chk1 (or dual inhibitors of Chk1 and Chk2). However, the effectiveness of ATR inhibition in combination with several different chemotherapeutic agents has also been demonstrated in the laboratory by several groups. The sensitization of cancer cells to chemotherapy by ATR inhibition is striking, and in some instances, much greater than the sensitization observed with Chk1 inhibition. In light of this finding, the pharmaceutical development of ATR inhibitors is a potentially useful and exciting strategy for cancer therapy.

## Abbreviations

5-FU: 5-fluorouracil
ATM: ataxia telengiectasia mutated
ATR: ataxia telengiectasia and Rad3-related
ATRIP: ATR-interacting protein
BLM: Bloom helicase
BRCA1: breast cancer (suppressor) 1
Chk1: checkpoint kinase 1
Chk2: checkpoint kinase 2
DSB: double-strand break
HR: homologous recombination
mTOR: mammalian target of rapamycin
NHEJ: non-homologous end joining
PIKK: phosphoinositide 3-kinase related kinase
PRD: PIK regulatory domain
RFC: replication factor C
RPA: replication protein A
ssDNA: single-stranded DNA
TOPBP1: topoisomerase binding protein 1
WRN: Werner’s syndrome protein
